# Phylogenetic Analysis of Alphacoronaviruses Based on 3c and M Gene Sequences Isolated from Cats with FIP in Romania

**DOI:** 10.3390/microorganisms12081557

**Published:** 2024-07-30

**Authors:** Ivona Popovici, Sophie Le Poder, Cristina-Mihaela Rîmbu, Cristina-Elena Horhogea

**Affiliations:** 1 Department of Public Health, Faculty of Veterinary Medicine, Ion Ionescu de la Brad Iași University of Life Sciences, 700490 Iasi, Romania; ivona.popovici@iuls.ro; 2 UMR Virologie, INRAe, ANSES, École Nationale Vétérinaire d’Alfort, 94704 Paris, France; sophie.lepoder@vet-alfort.fr

**Keywords:** coronavirus, cat, 3c gene, M gene, feline infectious peritonitis

## Abstract

Coronaviruses are widespread in mammals and birds, causing mostly digestive and respiratory problems. In cats, feline coronaviruses undergo mutations while replicating, giving rise to the fatal coronavirus causing the feline infectious peritonitis (FIP) disease. Several mutations in viral genes, among them 3c and M, are involved in the development of FIP. In order to study these viral shifts, samples of 43 organs, feces, and ascites collected from cats showing clinical signs of feline infectious peritonitis were tested, and the sequences obtained for the 3c and M genes were analyzed. The 3c gene nucleotides showed truncations commonly observed in feline infectious peritonitis virus. Additionally, the sequences corresponding to the 3c genes obtained from different organs of the same individual displayed high similarities, supporting the internal mutation theory. The analyses of the M gene and putative polypeptides showed similarities with canine coronaviruses, supporting the recombination theory between feline and canine coronaviruses. Infectious coronaviral strains are still challenging because of the difficulty in obtaining an effective vaccine for their prevention, and also because of the limited alternatives for therapy of FIP in cats.

## 1. Introduction

Coronaviruses are widespread in mammals and birds, causing mostly digestive and respiratory problems. For a while, the majority of the infections caused by coronaviruses were considered important for animal health, but since the SARS (Severe Acute Respiratory Syndrome) outbreak in 2003, followed by MERS (Middle East Respiratory Syndrome) in 2012, and culminating with the COVID-19 pandemic, the human health interest for these viruses has increased. Coronaviruses are capable of crossing the species barrier and this has been demonstrated, including the cases of emerging SARS-CoV and MERS-CoV in humans that have a bat coronavirus ancestor [1]. Phylogenetic analysis using the complete sequenced genome of coronaviruses identified in animals from different species has revealed close genetic relationships among some viral strains. This fact can be explained by these coronaviruses jumping interspecies from bats and birds, which serve as host reservoirs for most coronaviruses [2]. The etiologic agent of the COVID-19 pandemic is considered to have emerged from bats [3].

Genetic recombination occurs when different coronaviruses simultaneously infect the same host. For example, recombination between canine coronavirus and swine transmissible gastroenteritis virus has been identified by Decaro et al. (2009) in the organs of young dogs with acute gastroenteritis [4]. Recombinations between feline and canine coronaviruses have been reported by LePoder et al. (2013) [5] and Terada et al. (2014) in cats with feline infectious peritonitis [6].

Coronaviruses are enveloped and have a genome represented by a single-stranded positive RNA molecule consisting of 30 kb. The well-conserved structure of the genome comprises structural and accessory genes flanked by two noncoding 5′ and 3′ sequences. Two thirds at the 5′ region of the RNA molecule encode the proteins of the replication/transcription complex [7]. There are four structural genes called S, E, M, and N which encode, respectively, the spike, envelope, membrane, and nucleocapsid proteins. The accessory genes described in feline and canine coronavirus genomes are 3a, 3b, and 3c, located between the S and E genes and 7a and 7b, located downstream to the N gene. The role of these nonstructural genes is not yet understood, but changes in their sequence are involved in increasing virulence [8,9].

Two biotypes of feline coronaviruses (FCoV) have been described. Feline enteric coronavirus (FECV) is transmitted via the oral route, shows affinity for enterocytes, and causes mild enteritis. While this virus replicates, mutations can occur and lead to the appearance of the highly virulent feline infectious peritonitis virus (FIPV) that gains an affinity for macrophages. The viruses from this biotype spread systematically and cause lethal disease.

The mutations incriminated in tropism change, related to increased virulence, can affect especially the S, 3c, M, and 7b gene sequences. The detection of mutations occurring in the gene that encodes for the spike protein can help in discriminating between feline coronavirus strains found in the tissues of cats with FIP and feces of healthy cats [10]. The feline enteric coronaviruses are described to have intact 3c genes and an intact 3c protein involved in intestinal replication. Most of the FIPV strains presents mutations in the 3c gene that lead to truncation or inactivation of the corresponding protein which seems to be involved in systemic spreading [11]. Studies suggested that enteric feline coronavirus and feline infectious peritonitis virus can be distinguished by using the 3c gene as a pathotype marker [12]. The M gene encodes for the most abundant protein that is involved in virus budding and cell-mediated immunity. Five amino acid positions in the M protein were suggested by Brown et al. (2009) in a study to help in discriminating between FIPV and FECV [13]. Mutations in the 7b gene are also suspected to be involved in switching feline enteric coronavirus to feline infectious peritonitis virus, but results are contradictory [9].

Two genotypes of coronaviruses are also described in cats: FCoV type I and FCoV type II. Feline coronaviruses of both types have been incriminated in feline infectious peritonitis developing. The analyses of the type II FCoV sequence showed that it emerged from a double recombination between FCoV type I and CCoV (canine coronavirus) type II [14]. The sites of recombination are variable, given that the M and N gene sequences are considered to be the target sites for this process involved in the emergence of FCoV type II [6].

The appearance of feline infectious peritonitis virus is still a matter of debate. If some studies suggest that the lethal systemic feline infectious peritonitis virus appeared through mutations produced during the replication of feline enteric coronavirus [15], other studies suggest that there are virulent and avirulent strains circulating among cats [13].

In order to study the diversity of the coronavirus strains that emerge in FIP-suspected cats from Romania, the genetic material obtained from various pathological samples was tested by amplification, sequencing, and phylogenetic analysis of the 3c and M regions.

## 2. Materials and Methods

### 2.1. Samples

This study was conducted on 43 samples collected from four cats (M1–M4), all males. The felines were presented at the Clinics of the Faculty of Veterinary Medicine, from Ion Ionescu de la Brad Iași University of Life Sciences. The animals were suspected of the wet form of FIP based on their clinical signs, such as effusions in the abdominal cavity, dehydration, anorexia, and weight loss. Three of them were one year old and one kitten was four months of age. The animals were euthanized for ethical reasons with the approval of The Ethics Commission of the Faculty of Veterinary Medicine Iasi, within the University of Agricultural Sciences and Veterinary Medicine Ion Ionescu de la Brad Iasi, 839/27.09.2011.

Samples such as effusions (ascites fluid) and organs such as the small and large intestine, lymph node, spleen, liver, kidney, lung, heart, and pancreas were collected. The samples were stored in sterile tubes at −80 °C. Faecal samples were harvested using rectal swabs and suspended in PBS before storage.

### 2.2. Coronavirus Antibodies

Four ascites fluid samples were tested for the presence of coronavirus antibodies using an indirect immunofluorescence assay (indirect IFA). Briefly, 96-well plates containing porcine kidney cells culture were infected with TGEV (swine transmissible gastroenteritis virus). From serial dilutions of the samples (1/25, 1/25, 1/125, 1/625, 1/3125, and 1/16,000) prepared in PBS, 50 µL was added in each well and the plate was incubated at 37 °C for 30 min, then washed with PBS, before adding 50 µL goat anti-feline IgG labelled with fluorescein isothiocyanate (FITC) (AffiniPure Goat Anti-Cat IgG, Jackson, UK) diluted 1/100. The plates were incubated again for 30 min at 37 °C, washed with PBS, and examined using an Olympus immunofluorescence microscope. As a negative control, PBS was used.

### 2.3. RNA Extraction from Effusions and Faecal Samples

The coronavirus RNA was extracted from effusions and faecal samples using the QIAamp Viral RNA Mini kit from Qiagen (Hilden, Germany). Briefly, 140 µL of each sample was mixed with 560 µL of lysis buffer with RNA carrier and incubated for 10 min at room temperature. Following this step, 560 µL of ethanol was added and, after centrifugation, the viral RNA attached to the membrane of the Qiagen columns was recovered by washing with 50 µL of RNAse-free water.

### 2.4. RNA Extraction from Organ Samples

From the organs, the coronavirus RNA was extracted using an RNA-easy Mini kit from Qiagen. Tissue fragments of 30 mg were weighed, lysed, and homogenized in RLT (RNeasy Lysis Buffer)-β mercaptoethanol using a FastPrep24 instrument from MP Biomedicals. The lysate was centrifugated and the supernatant was mixed with ethanol. The mix was introduced in columns and centrifugated in order for RNA to bind to the column. After several washes, the RNA was recovered using 30 µL of RNase-free water. The extracted RNA for each sample was stored at −80 °C until amplification.

### 2.5. Amplification Using Real-Time RT-qPCR

Coronavirus presence in the samples was tested by real-time RT-qPCR using the following primers, 5′-GGC AAC CCG ATG TTT AAA ACT GG-3′ and 3′-CAC TAG ATC CAG ACG TTA GCT C-5′, which amplified a well-conserved region within the alphacoronavirus genus [16]. The amplification was conducted using the QuantiTect SYBR Green RT-PCR from Qiagen (Hilden, Germany) and a Light-Cycler 96 from Roche (Basel, Switzerland). The mix, prepared with 17 µL of amplification mix containing the two primers and 3 µL of RNA, was amplified using the annealing temperature of 52 °C.

### 2.6. Amplification Using RT-PCR

The sequences corresponding to the non-structural gene 3c segment were amplified using the sense 5′-CAAGTACTATAAAACGTAGAAGMAG-3′ and antisense 5′-CAGGAGCCAGAAGAAGACACTAA-3′ primers [11], annealed at a temperature of 50 °C. If the amplification result was negative, other primers, sense 5′-ATGGCATTGTGACAGCAACTG-3′ and antisense 5′-ATGAGAAGTTCTCACGGCTC-3′, annealed at 54 °C, were used in combination with the pair of primers described by Chang et al., 2010 [11].

For the amplification of the structural M gene, the sense 5′- TTGAACTAAACAAAATGAAG-3′ and antisense 5′-AATTATTACATATGGTGTAA-3′ pair was annealed at 55 °C. In the case of failing the amplification, the forward primer was replaced with 5′-TTTCCAGATATGTAATGTTCGG-3′, annealed at the same temperature.

The RT-PCR was performed using the One-Step RT-PCR kit from Qiagen. A mix of 24 µL containing reaction components from the kit and primers was prepared and 3 µL of RNA was added for each sample. The same amounts were used for the positive controls represented by porcine coronavirus transmissible gastroenteritis virus and feline coronavirus isolated on CRFK (Crandell Rees Feline Kidney) and the negative control represented by RNase-free water. The mixture was incubated at 50 °C for 40 min for reverse-transcription, then a temperature of 95 °C was required for 15 min for Taq DNA polymerase activation. The amplification was carried out by repeating 46 cycles, each consisting of denaturation at 94 °C for 40 s, followed by annealing at different temperatures depending on the primers used for 40 s and extension at 72 °C for 40 s. The last step of the RT-PCR was the final extension at 72 °C for 10 min.

The amplicons were stored at 4 °C, mixed with a dye, and submitted to electrophoresis using TBE buffer and 2% agarose gel containing ethidium bromide. The results were visualized using Gel Doc ^TM^ XR+ System from BIORAD. Using the MinEluteGel Extraction (QIAgen, Hilden, Germany), the cDNA was extracted from the electrophoresis gel and suspended in 20 µL of RNase-free water.

### 2.7. Sequencing

The amplicons were sequenced by Eurofins, Germany using the Sanger method and the sequences were introduced in GenBank, having the accession numbers OR772213–OR772226 and OR677898–OR677901.

### 2.8. Phylogenetic Analysis

The alignment of the amplified sequences was obtained using the Clustalw (https://www.ebi.ac.uk/jdispatcher/msa/clustalo?stype=dna) program. The aligned nucleotide sequences were used to construct phylogenetic trees with the MEGA (Molecular Evolutionary Genetics Analysis version 11) program. In order to analyse the corresponding amino acid chains, the nucleotide sequences were translated into putative proteins using the Transeq program (www.ebi.ac.uk/Tools/st/emboss_transeq). Nucleotide sequences corresponding to certain samples were too short, and for these samples there are no corresponding proteins. For the phylogenetic analysis of the 3c gene, the sequences with the GenBank accession numbers KP143512 [17], KP143507 [17], KP143508 [17], EU924791 [4], and HQ392470(UU19) [18] were used for comparison purposes, and for the M gene, the sequences with the GenBank accession numbers HQ738706 [19], HQ738725 [19], NP_058427 [20], MK507579, EU664100 [13], and HQ392470 (UU19) [18] were used (Table 1 and Table 2).

## 3. Results

### 3.1. Coronavirus Antibodies Titre

IFA revealed a titre of 1/625 coronavirus antibodies for cats M2 and M3, a titre of 1/3125 for cat M1, and a titre of 1/16,000 for cat M4. Even though antibodies’ presence in low titres is not consistent for FIP, high titres associated with ascites and pathological changes can be useful [21].

### 3.2. Amplification, Sequencing, and Phylogenetic Analysis

From the four felines (M1–M4), various pathological materials were harvested (Table 1). Using the real-time RT-PCR, feline coronavirus was detected in 31 samples from all four cats: all kidney, liver, lung, and small intestine (M1–M4) samples, three ascites (M1, M2, and M4), mesenteric lymph node (M1, M3, and M4) and spleen (M1, M2, and M4) samples, two heart (M2 and M3) and faecal (M3 and M4) samples, and one large intestine (M3) and pancreas sample (M3).

### 3.3. Results for the 3c Gene

In order to amplify the 3c gene in the samples that were positive for real-time RT-PCR, several pairs of primers were used. RT-PCR showed positive results for ten samples: two effusions (M2 and M4), three mesenteric lymph nodes (M1, M3, and M4), three small intestine (M2–M4) samples, and one pancreas (M3) and one faecal sample (M3) (Table 3). The 3c amplicons sequenced in order to obtain the corresponding nucleotide sequences succeeded for the cat M1 lymph node, cat M2 ascites and small intestine, cat M3 pancreas, faeces and lymph node, and cat M4 ascites. The sequencing failed for the rest of the 3c amplicons.

The nucleotide sequence for the cat M1 lymph node virus was 430 bases long at the 3′ end of the 3c gene. It presented 14 mismatches and one deletion when compared with the UU19 strain that led to six amino acid differences between the two viruses. The feline coronavirus UU19 strain was used, as it has been completely sequenced and the complete genome is accessible in GenBank with the accession number HQ392470.

For the 3c gene from the cat M2 ascitic fluid, we obtained a 471-nucleotide sequence in the 5′ end comprising the 25,147–25,611 positions. At the start codon, there was a point mutation where an A base was replaced by a T base. An insertion of six nucleotides in position 118 was observed also in the 3c gene of the coronavirus from the cat M2 ascites. This insertion led to an insertion of two amino acids in the putative 3C protein. The nucleotide sequence for the cat M2 intestine was 432 bases long and corresponded to the 25,455–25,885 positions when compared with the UU19 strain. The deletion of two nucleotides in the amplified region of the cat M2 intestine was observed in position 370–371 of the 3c gene sequence and led to a frameshift mutation and formation of numerous stop codons. The 3′ end of the gene failed to amplify in this case.

Similarities between the 3c gene of the coronaviruses in the cat M2 samples could not be analyzed because the amplified sequences occupied different positions.

When we compared the 3c sequences obtained for cats M1 and M2 (which came from the same environment), we did not observe the same mismatches in the samples compared with the UU19 strain.

**Figure 1:**
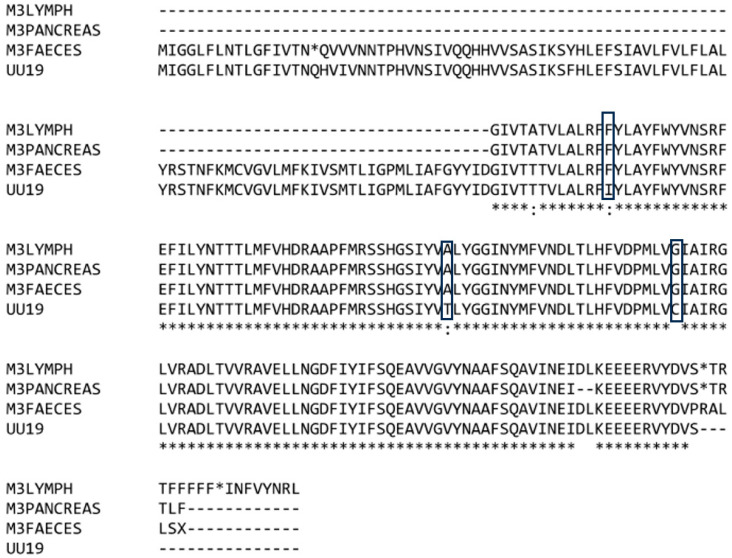
Clustalw alignment of the 3c proteins obtained from different samples collected from the same individual (M3) showing identical amino acids in the same positions for the coronavirus sequences compared with the coronavirus strain UU19. The “*” at the bottom shows where the aminoacids are identical in all the samples; the “:” indicates the positions where there are differences between aminoacids of the analyzed samples. The “*” in the sequence of aminoacids indicates a stop codon. The “-” was used when no aminoacid was available (due to deletions). The box indicates the amino acids of interest for the 3c protein analyses.

All the 3c gene nucleotide sequences obtained from the lymph node, pancreas, and fecal samples collected from the cat M3 presented nine identical substitutions when compared with the UU19 FCoV strain. When translated into proteins, three identical amino acids were present at the same positions in all three samples from the cat M3 case, different from the amino acids in the corresponding positions of the UU19 strain (Figure 1). This result is in accordance with the internal mutation theory, wherein the viruses from different tissues of the same individual share common mutations because they arise from a common ancestor, as described in other studies [15]. The high identity of the nucleotide sequences shared by viruses in different tissues of the same individual and fecal viruses are in concordance with other studies. The identity of the 3c gene nucleotides for tissue and fecal samples collected from the same individual varied between 95.58% to 99.09% in some studies [12], and was higher than 99% in others [22].

The 3c sequences from organ samples presented the same six mismatches when compared with the 3c sequence from the fecal sample of the same individual (M3). For the fecal sample, we obtained a 727-nucleotide sequence with an insertion of 13 nucleotides at the 3′ end when compared with UU19. The fecal sample sequence encoded for only 16 amino acids due to the presence of a premature stop codon induced by the mutation of one nucleotide (C instead of T) in position 49 of the gene. The detection of a truncated 3C gene in feces collected from FIP individuals was noticed in other studies [23]. The shedding of feline coronaviruses with truncated 3c genes supports the horizontal transmission of FIPV, in considering that non-coding mutations in the 3c gene are specific for feline infectious peritonitis virus. The horizontal transmission theory sustains that virulent strains of feline coronaviruses can be transmitted to other cats [24].

For the organs of cat M3, we did not succeed in amplifying the whole 3c gene but only a part of it. The 431-nucleotide sequence at the 3′ end of the gene 3C between the 25,455–25,885 positions that we succeeded in amplifying for the pancreas and lymph node samples of cat M3 were identical. These findings are in accordance with those described by Dye and Siddell (2007) [25] in a study in which FCoV strains collected from different tissue samples of the same individual were identical. The same situation is described by Chang et al. (2010) for the 3c nucleotide sequences detected in the gut and internal organs, which presented more similarities with each other when compared with those in other cats with FIP [11].

**Figure 2:**
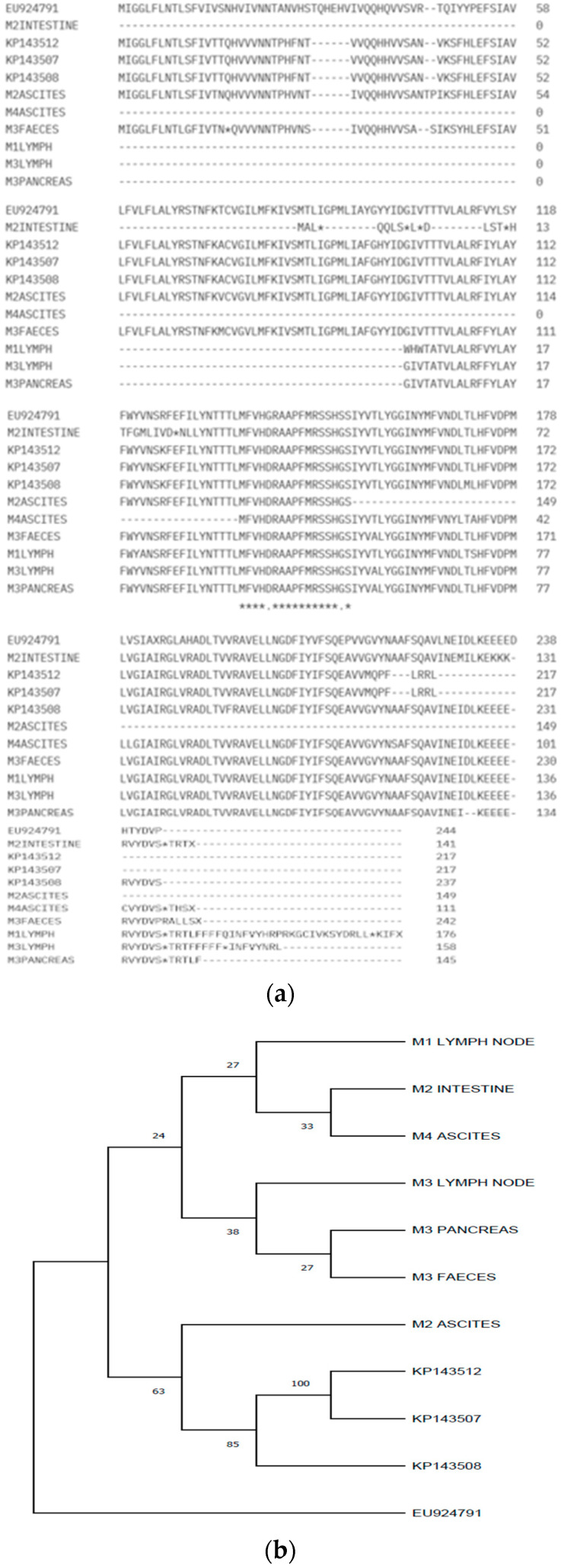
(**a**) The predicted 3c polypeptide alignment. The “*” at the bottom shows where the amino acids are identical in all the samples; the “.” indicates the positions where there are differences between amino acids of the analyzed samples. The “*” in the sequence of amino acids indicates a stop codon. The “-” was used when no amino acid was available (due to deletions). (**b**) Phylogenetic maximum parsimony tree obtained using a gapless alignment of the 3c gene corresponding polypeptides. The 3c canine coronavirus polypeptide (GenBank accession number EU924791) was used as an outgroup. The percentage of replicate trees in which the associated taxa clustered together in the bootstrap test (1000 replicates) are shown next to branches. Evolutionary analysis was conducted in MEGA11.

For cat M4, the sequencing succeeded only for the ascites sample, where 390 bases were obtained (25,496–25,885) at the 3′ end of the 3C protein. There were 16 mismatches and three deletions in the amplified M4 ascites virus when compared with the UU19 strain. The phylogenetic analysis (Figure 2) shows the diversity of the strains that appeared due to mutations.

The phylogenetic tree for the 3c gene was constructed using a gapless alignment of the corresponding polypeptides with the Maximum Parsimony method and a bootstrap confidence value of 1000 replicates. For the phylogenetic tree, we used a 3c canine coronavirus (GenBank accession number EU924791, from a Decaro et al., 2009 [4] study) as an outgroup, as described by Chang et al., 2010 [11]. The 3c gene’s corresponding polypeptides with the GenBank accession numbers KP143507, KP143508, and KP143512 from a Lewis et al., 2015 [17] study were used to form a separate cluster, as expected for the coronaviruses that come from the same multi-cat environment or household [11]. Some of the sequences in our study formed separate strains, like M2, showing the diversity of mutations that can appear.

### 3.4. Results for M Gene

Real-time RT-PCR positive samples were further tested by RT-PCR for M gene amplification. We obtained 104- to 374-nucleotide sequences, showing positive results for 14 samples: three effusions (M1, M2, and M4), three mesenteric lymph nodes (M1, M3, and M4), one large intestine (M3), two small intestine (M2 and M3), one pancreas (M4), one liver (M3), one lung (M3), one kidney (M2), and one feces (M3) sample (Table 4).

**Figure 3:**
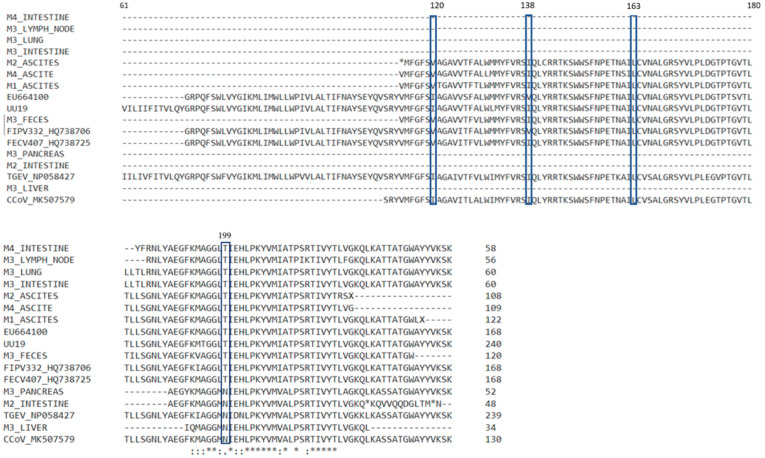
Alignment with ClustalW of amino acid polypeptide sequences corresponding to the M gene of coronaviruses identified on different samples with the five sites incriminated in feline coronavirus pathogenicity. The “*” at the bottom shows where the amino acids are identical in all the samples; the “.”and “:” indicates the positions where there are differences between amino acids of the analyzed samples. The “*” in the sequence of amino acids indicates a stop codon. The “-” was used when no amino acid was available (due to deletions).

From the 14 nucleotide sequences, we succeeded in amplifying for the M gene, only 11 were successfully sequenced: the cat M1 ascites, cat M2 ascites and small intestine, cat M3 large intestine, liver, lung, lymph node, pancreas, and feces, and cat M4 ascites and small intestine. The sequences presented mutations such as substitutions and deletions that modified the frameshift, leading to missense and nonsense amino acids in the putative proteins (Figure 3). The M2 ascites M gene presented one stop codon in position 113, corresponding to the UU19 M protein. There were two stop codons in the hypothetical M2 intestine M gene. In this gene, the deletion of two amino acids was also noticed. In the M4 intestine, a premature stop codon was present, followed by a modified sequence of amino acids. Amino acid sequences in the corresponding/putative M proteins were compared with feline enteric coronavirus, feline peritonitis virus, canine coronavirus, and TGEV sequences available in GenBank. Three sequences (M2 intestine, M3 liver, and M3 pancreas) showed an identity of 96.23%, 99.03%, and 98.37%, respectively, with canine coronaviruses. The higher homology between three nucleotide sequences corresponding to the M gene with the canine coronaviruses compared to feline coronaviruses can be explained by the recombination between canine and feline coronaviruses.

Brown et al. in 2009 described five informative aa sites incriminated in the making the difference and discriminating between the enteric strains and systemic strains of coronavirus at positions 108, 120, 138, 163, and 199 [13]. We compared and analyzed the membrane proteins with respect for the five mentioned sites (Figure 3).

Because our sequences were shorter, we failed to obtain the nucleotide sequences for the 5′ end of the M gene and did not obtain the amino acids at position 108. The position 120 in the M protein corresponds to isoleucine for the FIPV strains, and to isoleucine or valine in the case of FECV strains, according to the Brown et al. study from 2009 [13]. We obtained the amino acids corresponding for the position 120 from three ascites samples, and this position was occupied by valine. This is in disagreement with the study in which valine in position 120 was found in the feline coronaviruses from cats without clinical FIP, while in our study it was found in ascites samples from cats with FIP. For the canine coronavirus (CCoV_MK507579) used as a reference, this position was also occupied by valine. In the position 138 there was isoleucine in all our available sequences, which is in accordance with other studies [13].

Further analyses of the putative proteins showed that in the 138, 163, and 199 positions, the M3 liver, M3 pancreas, and M2 intestine samples presented the same amino acids as the canine coronavirus strain. The amino acids in these positions were different for the other samples in our study and the feline coronaviruses accessed from GenBank (Figure 3). The nucleotide sequence for these samples also presented the highest homology with the canine coronavirus when blasted. Using the phylogenetic analyses, the sequences corresponding to the M gene for samples of M3 pancreas, M3 liver, and M2 intestine were segregated in different clusters, showing more similarities to the canine coronavirus strain CCoV MK507579 and porcine coronavirus TGEV NP-58427.

## 4. Discussion

From 31 real-time RT-PCR coronavirus-positive samples, we succeeded in amplifying the 3c gene in only 10 samples, but obtained only seven nucleotide sequences for these. When compared with coronavirus strains from GenBank, our sequences were more similar to type I feline coronavirus, this type being more common, as studies suggest [26]. Type II feline coronavirus arises from the recombination of type I feline coronavirus and canine coronavirus [14]. Further studies should be made in order to classify our FCoV into type I and II. The 3c sequences obtained from different organ samples collected from the same cat were more similar to each other when compared with the 3c sequences from other cats in the study. Two cats (M1 and M2) belonged to the same owner and shared the same environment, but between their 3c sequences, no similarities were noticed. These findings are in accordance with other studies [12,27] that sustain that the 3c gene mutations are unique to each cat. The incomplete 3c gene sequences obtained from the organs (M3 pancreas and M3 lymph node) of the same cat showed a 100% homology. The same aspect was observed by Dye and Siddell when identical coronavirus strains were obtained from the jejunum and liver of a cat with FIP [25]. The 3c genes in the three samples (M3 lymph node, M3 pancreas, and M3 feces) collected from the same cat were clustered together in the phylogenetic tree. This situation supports the internal mutation theory [15].

The 3c gene sequences that we obtained presented missense mutations, deletions, and insertions leading to inactivated or truncated 3c proteins. A premature stop codon was generated by a mutation that involved a single nucleotide in the sequence of a fecal sample (M3). In five samples, the 5′ end of the 3c gene could not be amplified, and for four samples, the 3c gene could not be amplified at all; thus, we concluded that it was more difficult to identify the 3c gene compared with the M gene, for which we succeeded in obtaining 11 nucleotide sequences. The difficulty of amplifying the 3c gene was observed and explained by some authors [8] who suggested the low quantity of 3c and also the variability at the primers’ binding site. The mutations that generate truncation and inactivation of the 3c protein of FIPV do not affect the viral systemic replication, compared with FECV, which requires an intact 3c gene to replicate in the intestinal tract [27]. In this way, the deadly FIPV strain arises from FECV. The feline enteric coronavirus with an intact 3c gene replicates in enterocytes where feline infectious peritonitis virus has affinity and can replicate in monocytes and macrophages that spread throughout the organism. Therefore, mutations in the 3c gene are corelated with increased virulence and FIPV development [28]. Some authors suggested, after detecting FIPV in the feces of a cat with feline infectious peritonitis, that prior to death, FIPV can replicate in the gut [29]. The presence of the of truncated 3c genes in feces was reported in other studies too [27]. This aspect was also observed in our study, wherein we succeeded in amplifying the 3c gene from feces, and this showed similarities with other 3c sequences of FCoV from pancreas and lymph node samples of the same individual (cat M3). When phylogenetic analysis was performed, the 3c gene from feces formed a separate branch with other 3c sequences identified in the pancreas and lymph node of the same individual (Figure 2b).

**Figure 4:**
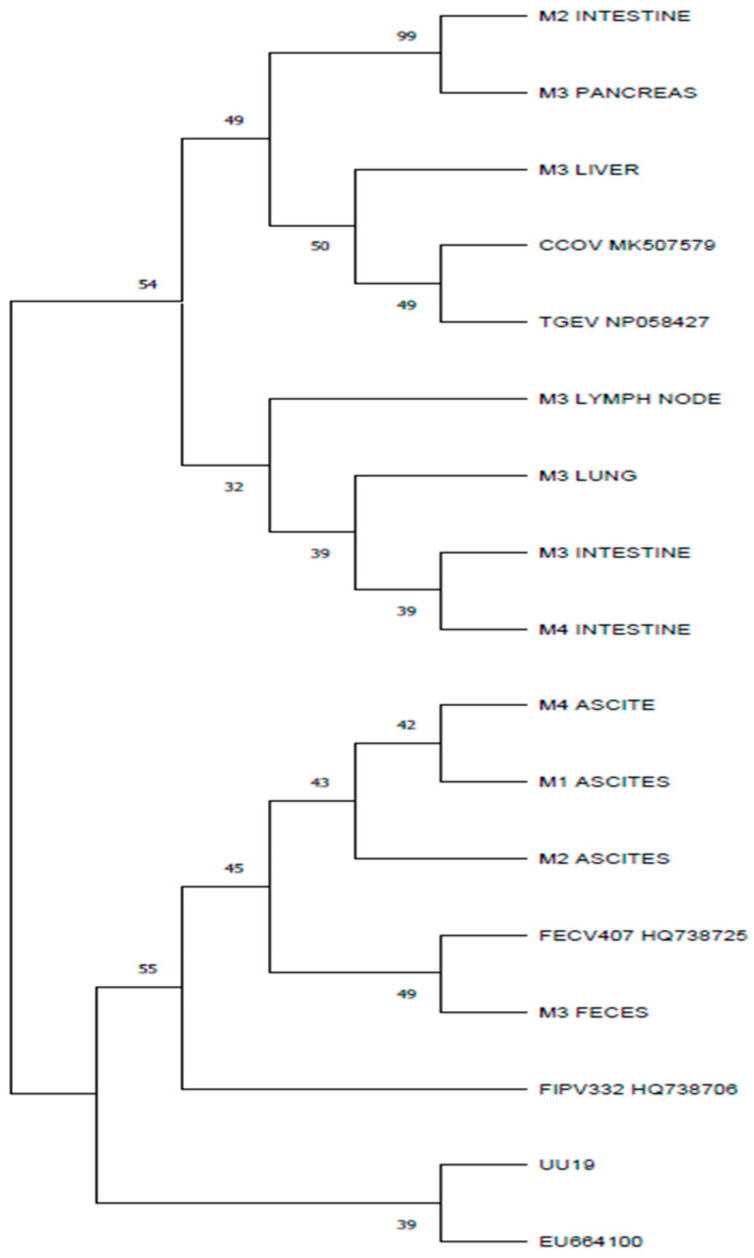
Phylogenetic tree based on amino acid polypeptides corresponding to M proteins, constructed using the maximum parsimony obtained using a gapless alignment of the M gene corresponding polypeptides. The percentage of replicate trees in which the associated taxa clustered together in the bootstrap test (1000 replicates) are shown next to branches. Evolutionary analysis was conducted in MEGA11.

Studies showed that the recombination sites between feline and canine coronaviruses can occur at the M gene [6]. The presence of M gene nucleotide sequences with high homology to either feline or canine coronavirus observed in different organs of the same individual is in accordance with other studies in which the presence of different coronavirus strains in the same organism is described [23]. This situation was observed in two cats (M2 and M3) from the four in this study. The phylogenetic analysis showed that the M genes from the M2 intestine sample and from the M3 pancreas showed more similarities with CCoV than with FCoV. However, the M genes from the M3 feces samples and from the M2 ascites showed more similarities and clustered into separate branches with FCoVs (Figure 4). The presence of the stop codon was observed in three of the M proteins: the M2 ascites, M2 intestine, and M4 intestine samples. The presence of stop codons in the M gene was described in other studies [12]. The M gene encodes the structural matrix protein, the most abundant envelope protein in coronaviruses. A truncated matrix protein may interfere with the function of the virion, but some authors [12] suggest that the sequence encoding for the matrix protein is involved in recombination that increases coronaviruses’ genetic diversity and leads to more virulent strains (their genome may be involved in recombination, increasing the genomic diversity and leading to more virulent strains). Coronaviruses can be transmitted between animal species, thus permitting recombination and leading to new viruses.

## 5. Conclusions

The results presented in this study refer to coronaviral strains that circulate in and between felines in Romania. The findings in our research regarding the 3c gene and M gene support the internal mutation theory and the recombination between FCoV and CCoV theory. Due to mutations resulting during replication, different strains of feline coronaviruses can appear in the same individual, some of them leading to new characteristics that increase virulence. Infectious coronaviruses are still challenging because of the difficulty in obtaining an effective vaccine for effectiveness in prevention, and also because of ineffective treatments and the high death rate among FIP cats.

## Figures and Tables

**Table 1:** Reference sequences used for the phylogenetic analysis of the M gene.

Coronavirus Strain	GenBank Accession No	Year of Collection	Host	Source
FIPV332	HQ738706	2009	cat	[19]
FECV407	HQ738725	2010	cat	[19]
TGEV_NP058427	NP_058427	Tissue culture adapted clone of the TGEV Purdue strain	pig	[20]
CCoV_MK507579	MK507579	2018	dog	Not available
EU664100 (FIPV)	EU664100	2004	cat	[13]
UU19 (FECV-complete genome)	HQ392470	2007	cat	[18]

**Table 2:** Reference sequences used for the sequence and phylogenetic analysis of 3c gene.

Coronavirus Strain	GenBank Accession No	Year of Collection	Host	Source
Feline Coronavirus isolate 26 M	KP143512	2013	cat	[17]
Feline coronavirus isolate 27C	KP143507	2013	cat	[17]
Feline coronavirus isolate 28O	KP143508	2013	cat	[17]
UU19 (FECV-complete genome)	HQ392470	2007	cat	[18]
Canine coronavirus strain 119/08	EU924791	2008	dog	[4]

**Table 3:** Results of 3c gene amplification and sequencing.

Cat	M1			M2			M3			M4		
Sample	Real-Time-RT-PCR	RT-PCR	Sequencing	real-Time-RT-PCR	RT-PCR	Sequencing	Real-Time-RT-PCR	RT-PCR	Sequencing	Real-Time--RT-PCR	RT-PCR	Sequencing
ascites	+	-	-	+	+	471 pb	NA	NA	NA	+	+	390 pb
feces	NA	NA	NA	NA	NA	NA	+	+	727 pb	+	-	-
heart	NA	NA	NA	+	-	-	+	-	-	NA	NA	NA
kidney	+	-	-	+	-	-	+	-	-	+	-	-
large intestine	NA	NA	NA	NA	NA	NA	+	-	-	NA	-	-
liver	+	-	-	+	-	-	+	-	-	+	-	-
lung	+	-	-	+	-	-	+	-	-	+	NA	NA
lymph node	+	+	430 pb	NA	NA	NA	+	+	442 pb	+	+	-
pancreas	NA	NA	NA	NA	NA	NA	+	+	405 pb	NA	NA	NA
small intestine	+	-	-	+	+	432 pb	+	+	-	+	+	-
spleen	+	-	-	+	-	-	-	-	-	+	NA	NA

+ (positive); - (negative); NA (not available).

**Table 4:** Results of M gene amplification and sequencing.

Cat	M1			M2			M3			M4		
Sample	Real-Time-RT-PCR	RT-PCR	Sequencing	Real-Time-RT-PCR	RT-PCR	Sequencing	Real-Time-RT-PCR	RT-PCR	Sequencing	Real-Time- RT-PCR	RT-PCR	Sequencing
ascites	+	+	374 pb	+	+	373 pb	NA	NA	NA	+	+	368 pb
feces	NA	NA	NA	NA	NA	NA	+	+	382 pb	+	NA	NA
heart	NA	NA	NA	+	NA	NA	+	NA	NA	NA	NA	NA
kidney	+	NA	NA	+	+	-	+	NA	NA	+	NA	NA
large intestine	NA	NA	NA	NA	NA	NA	+	+	201 pb	+	NA	NA
liver	+	NA	NA	+	NA	NA	+	+	104 pb	+	NA	NA
lung	+	NA	NA	+	NA	NA	+	+	200 pb	+	NA	NA
lymph node	+	+	-	NA	NA	NA	+	+	189 pb	NA	+	-
pancreas	NA	NA	NA	NA	NA	NA	+	+	194 pb	+	NA	NA
small intestine	+	NA	NA	+	+	181 pb	+	NA	NA	+	+	263 pb
spleen	+	NA	NA	+	NA	NA	-	NA	NA	NA	NA	NA

+ (positive); -(negative); NA (not available).

## Data Availability

The original contributions presented in the study are included in the article, further inquiries can be directed to the corresponding authors.
